# Case Report: Autoimmune Pemphigus Vulgaris in a Patient Treated With Cemiplimab for Multiple Locally Advanced Cutaneous Squamous Cell Carcinoma

**DOI:** 10.3389/fonc.2021.691980

**Published:** 2021-08-23

**Authors:** Rosalba Buquicchio, Valentina Mastrandrea, Sabino Strippoli, Davide Quaresmini, Michele Guida, Raffaele Filotico

**Affiliations:** ^1^Dermato-Oncology Unit, Istituto di Ricovero e Cura a Carattere Scientifico (IRCCS) Istituto Tumori “Giovanni Paolo II”, Bari, Italy; ^2^Melanoma and Rare Tumors Unit, Istituto di Ricovero e Cura a Carattere Scientifico (IRCCS) Istituto Tumori “Giovanni Paolo II”, Bari, Italy

**Keywords:** Pemphigus vulgaris, desmoglein, immune check-point inhibitors, cemiplimab, anti-programmed-death-1, cutaneous squamous cell carcinoma

## Abstract

**Background:**

Pemphigus vulgaris (PV) is a rare and severe autoimmune blistering disorder affecting the skin and mucous membranes, characterized by the production of autoantibodies against two desmosomal adhesion proteins, desmoglein 1 and 3. In patients with advanced squamous cell carcinoma of the skin unfit for surgery and radiotherapy, immune check-point inhibitors, including the anti-Programmed Death-1 (PD-1) agent cemiplimab have been successfully employed proving relevant clinical outcomes. Cemiplimab is a monoclonal antibody capable of inhibiting PD-1 signalling that has recently been approved for the treatment of patients with metastatic or locally advanced cutaneous squamous cell carcinoma. Although the peculiar setting of advanced CSCC involving elderly patients, rare and unusual skin immune-related adverse events such as PV could be observed in cemiplimab treated patients.

**Case Report:**

A 95-year-old man without a history of autoimmune disease was treated with cemiplimab for multiple and advanced squamous cell carcinomas of the head obtaining a complete response to therapy. After seven cycles of cemiplimab administered every 21 days, the patient developed a mucocutaneous blistering eruption. Clinical diagnosis of PV was suspected on the basis of the diffuse involvement of trunk and extremities with large blisters and necrotic eschar. It was carried out an ELISA test, that showed high level of circulating antibodies against desmoglein 1, thus confirming the diagnosis of PV. For this reason, cemiplimab infusion was discontinued and complete resolution of skin lesions was obtained using oral prednisone 0,8 mg/kg/daily for four weeks. Once remission was achieved, a maintenance dose of 10 mg/day was administered, observing a good control of bullous disease and low value of desmoglein 1. Response to CSCC persisted also during cemiplimab discontinuation, until obtaining a complete remission still persisting at 9 months after the last cycle of therapy.

**Conclusion:**

The case we observed is the first description of PV revealed from cemiplimab therapy, thus suggesting that cemiplimab could allow the arise of underlying autoimmune PV, through a mechanism both T and B-cell-mediated.

## Introduction

Pemphigus vulgaris (PV) is a rare and severe autoimmune blistering disorder that affects the skin and mucous membranes (1). PV is characterised by the production of pathogenic autoantibodies directed against two desmosomal adhesion proteins, desmoglein Dsg1 and Dsg3 (also known as DG1 and DG3), which are present in the skin and mucosae (1). The binding of autoantibodies to Dsg proteins induces a separation of neighbouring keratinocytes *via* a process known as acantholysis, leading histologically to intraepidermal blisters, and clinically to blisters and erosions on the epithelium of the mucous membranes and/or the skin (1). Since the pathophysiology is driven by an autoimmune process, autoantibodies are the basis of diagnostic investigations and treatment strategies.

Cutaneous squamous cell carcinoma (CSCC) is a highly incident skin cancer that is often characterised by multifocal presentation and high rates of local recurrence after surgical excision. Advanced CSCCs include a small number of metastatic patients and more frequent cases of locally advanced disease unfit for both surgery and radiotherapy, which can only be treated by systemic therapy (2, 3). Immunotherapy with anti-programmed death ligand-1 (PD-1) agents is the gold standard in all current guidelines, and cemiplimab is the only recently approved anti-PD-1 antibody in Italy (4–6).

The increasing use of checkpoint inhibitors for the treatment of advanced skin cancers, with positive response to therapy, is related to an increase in adverse skin reactions. Among such side effects, diseases with autoimmune pathogenic mechanisms have also been described (7).

We report the first case of a patient who developed severe PV during cemiplimab therapy for locally advanced CSCC.

## Case Description

A 95-year-old man with no known history of autoimmune disease developed widespread mucocutaneous blistering during cemiplimab therapy, which was administered for multiple and advanced squamous cell carcinomas of the head.

**Figure 1:**
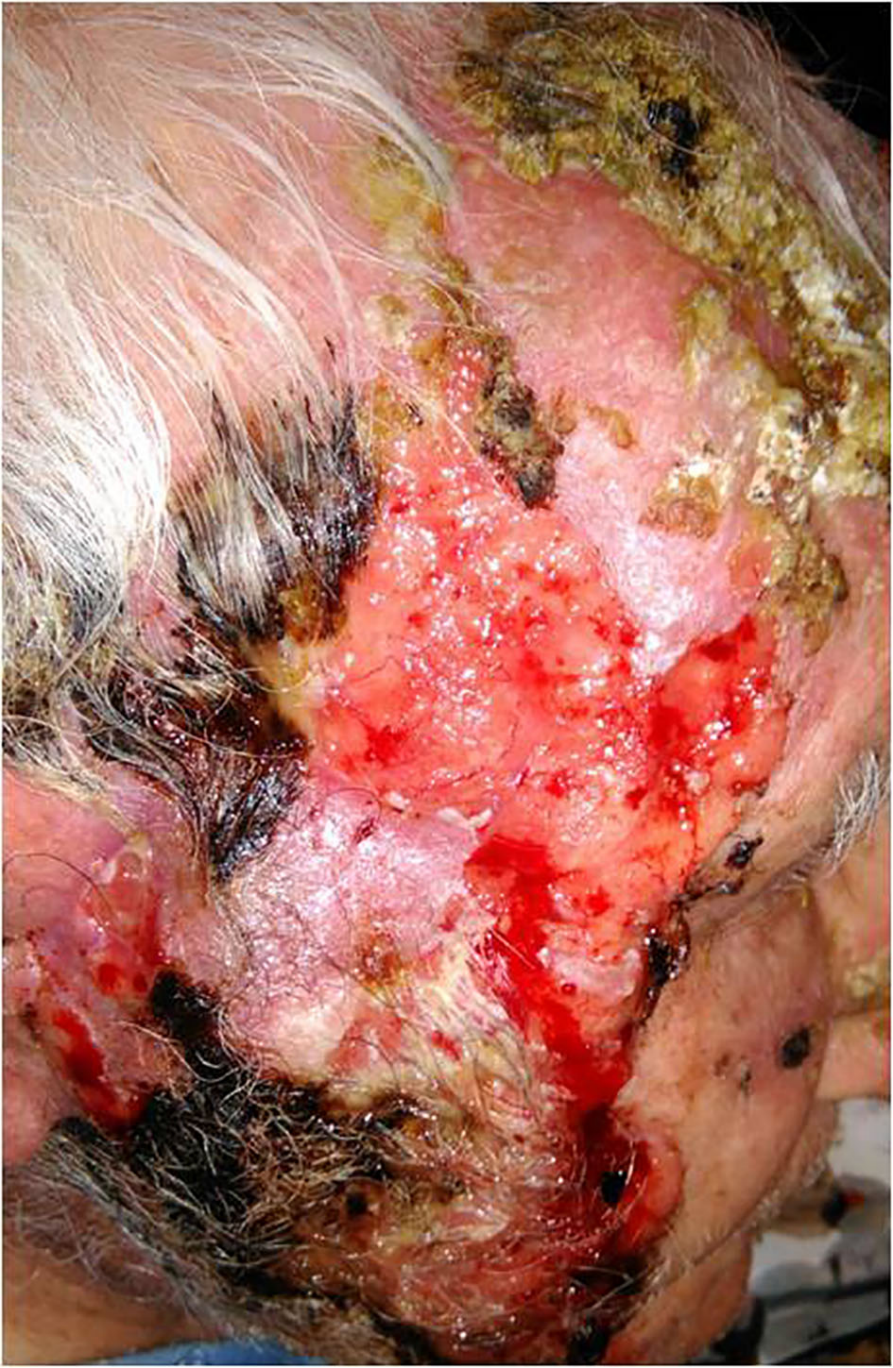
Locally advanced locally cutaneous squamous cell carcinoma of the face extending to the zygomatic area close to the lower eyelid, the right cheek and the mandibular area, characterized by necrotic ulcerated areas and infiltration of subcutaneous tissue.

He had undergone multiple resections of CSCCs of the head and neck region, three of which were performed during the preceding year. Over the last 6 months, he exhibited a local relapse in the right parotideal area, with a rapidly growing, ulcerated, and bleeding lesion extending to the zygomatic area close to the lower eyelid, the right cheek, and the mandibular area (**Figure 1**). Histological examination of the lesion showed infiltration of subcutaneous tissue, with a high proliferative rate (Ki-67, 90%) associated with wide necrotic and ulcerated areas, thus confirming the clinical impression of CSCC. Clinically, other smaller but similar lesions were localised in the patient’s left zygomatic area, the left ear, and upper limbs. Computed tomography (CT) scan was negative for regional or distant metastases. The patient was initiated on immunotherapy with cemiplimab (350 mg flat dose every 3 weeks) in February 2020. Soon after the first cycle, the lesions became non-ulcerated and progressively thickened with crust evolution. The treatment was well tolerated, except for occasional intermittent grade 1 pruritus after cycle 5, which was responsive to anti-histamines. After completion of seven cycles, ubiquitous splinter lesions appeared on the patient’s body, first occurring in the lower limbs, then extending to the trunk and upper limbs, alongside similar lesions in the mucosa of the oral cavity.

Due to the onset of this severe skin reaction, classified as grade four (G4), cemiplimab therapy was permanently discontinued. Suspecting an adverse reaction to cemiplimab, the patient was administered systemic steroid therapies, with incomplete remission of the dermatosis. However, the patient relapsed shortly after dose reduction.

**Figure 2:**
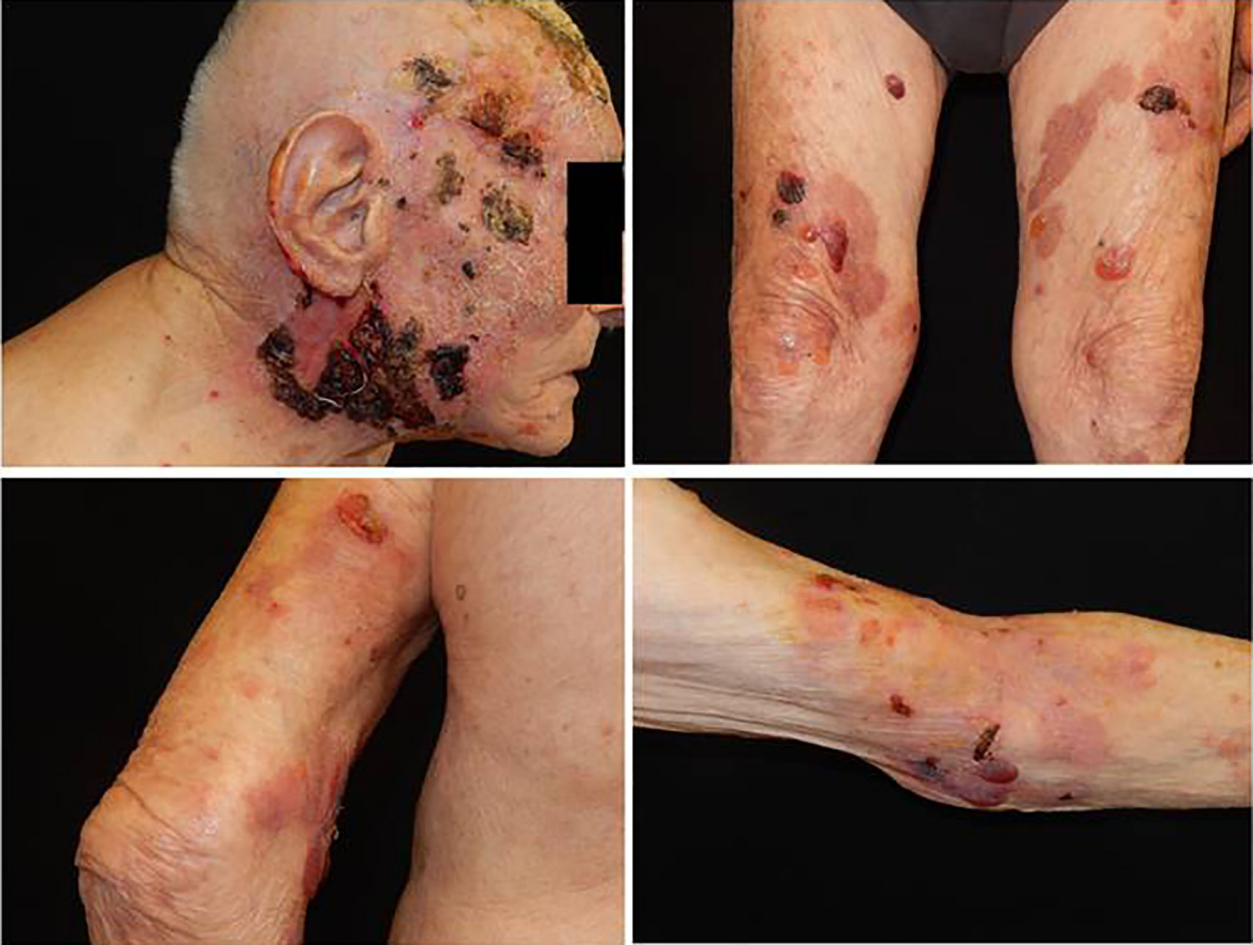
A large necrotic haemorragic eschar on the head (face and scalp), with ulcers and serohematic scars associated with blisters over skin, with serum content, localized on the trunk and extremities.

At our department, clinical examination of the patient revealed diffuse involvement of the trunk and extremities with large blisters over the skin, with serum content, excoriations, and large necrotic haemorrhagic eschar on the head (face and scalp) (**Figure 2**). The patient had multiple comorbidities, such as arterial hypertension, hypertensive cardiomyopathy, and dyslipidaemia. The dermatological history was negative for a previously arisen bullous eruption. The patient had no underlying skin or autoimmune disorders, no recent exposure to light or radiation, and was not under any new medications. The first clinical differential diagnosis included paraneoplastic pemphigus (PNP), PV, and bullous pemphigoid (PB).

Due to the possibility of an autoimmune bullous disease, we suggested testing for serum IgG autoantibodies against desmoglein 1, 3, BP180, and BP230. Enzyme-linked immunosorbent assays (ELISA) showed a positive reaction with desmoglein (Dsg) 1: index value 28 U/ml; (reference positive >20 U/ml), but not with Dsg 3, BP 180, or BP 230. Based on clinical features and laboratory investigations, a diagnosis of PV was made. Consequently, the patient received treatment with prednisone 0.8 mg/kg/daily for 4 weeks and topical steroids, with resolution of skin lesions. Oral steroid therapy dose was gradually narrowed down to 25 mg per day, resulting in complete remission of the dermatosis.

**Figure 3:**
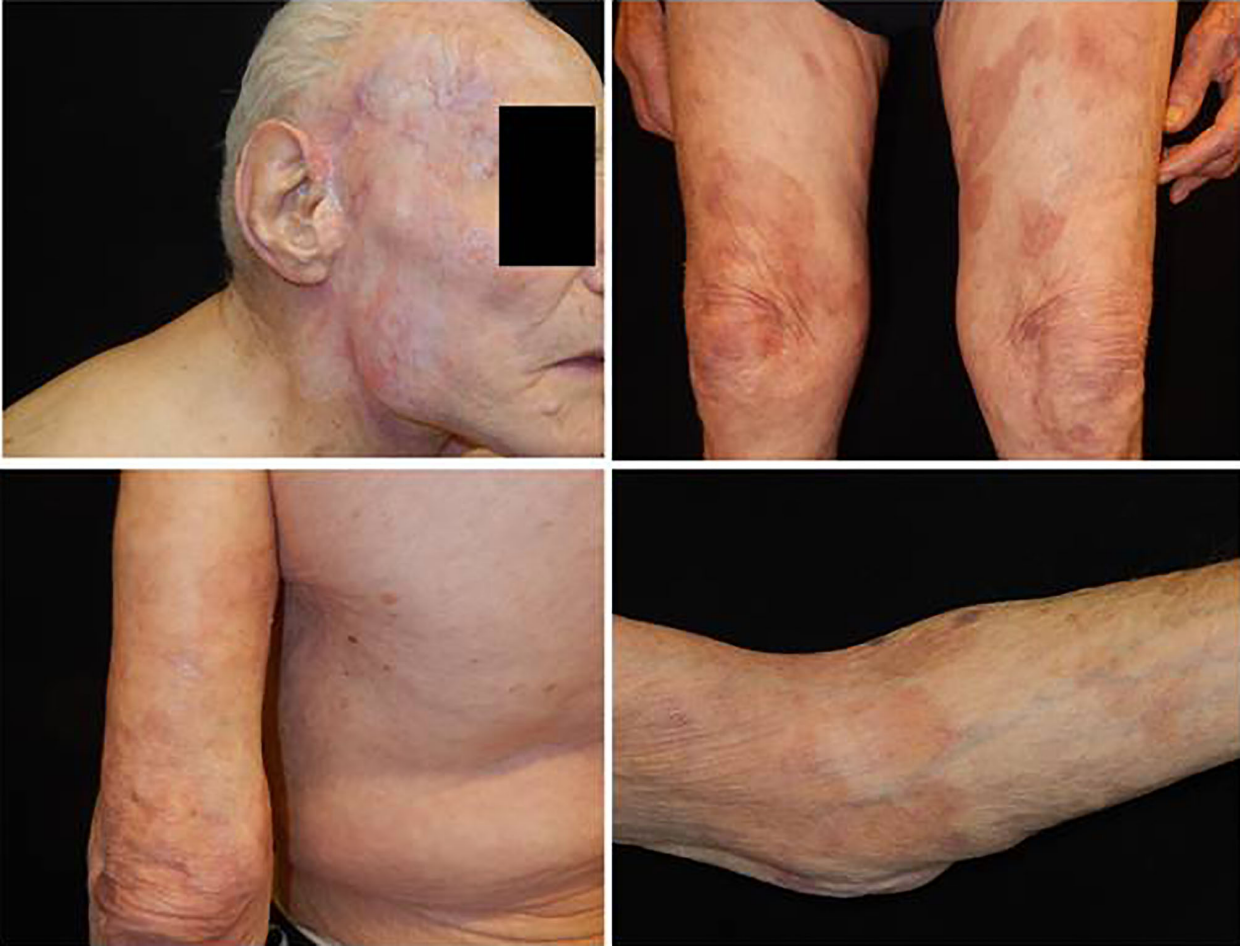
Complete remission of cutaneous squamous cell carcinoma on the head, and absence of bullous lesions on the trunk and the extremities.

Approximately 5 months after the first observation, the patient showed significant recovery, and there was no recurrence of dermatosis (**Figure 3**). Therefore, we reduced the dosage of prednisone to 12.5 mg per day.

## Discussion

A wide range of immune-related skin disorders have been observed in patients treated with immune checkpoint inhibitors (7). Immune checkpoint inhibitors (ICIs) represent a new class of anticancer agents. They belong to a class of drugs implicated in the inhibition of programmed cell death receptor 1 (nivolumab, pembrolizumab, and cemiplimab), PD-1 (atezolizumab, avelumab, and durvalumab), and cytotoxic T-lymphocyte-associated protein 4 (ipilimumab) (4). By blocking cytotoxic T-lymphocyte-antigen 4 (CTLA-4) and programmed cell death receptor 1 (PD-1) or its ligand; programmed death ligand 1 (PDL-1), they release negative inhibitory control of the immune system. This reverses T-cell suppression, thereby inducing an antitumor response (8).

Despite their efficacy against many malignancies, immunomodulatory antibodies non-specifically activate the immune system, which can lead to a new spectrum of immune-related adverse events (irAEs), like dermatologic toxicities (9). irAEs are described as a consequence of immune reactivation, with an unpredictable inflammatory response, loss of self-tolerance, and development of autoimmunity (10).

The precise mechanisms underlying the development of immune-related adverse events have not been fully elucidated but are postulated to be largely T cell-mediated. Monoclonal antibodies that target PD-1/PDL-1 pathways may induce immune-mediated adverse events possibly related to a reduction in regulatory T cells, leading to increased T-cell activation, B-cell proliferation, and synthesis of autoantibodies (7).

Cutaneous adverse events are reported in approximately 30%–40% of patients receiving immunotherapy. Their clinical expression can lead to pruritus, maculopapular rash, pigmentary changes, eczematous dermatitis, psoriasis, lichenoid dermatitis, vitiligo, and other inflammatory skin diseases (11). Autoimmune blistering disorders represent approximately 1% of cutaneous immune-related adverse events (12).

Previous literature has also reported occurrence of the present anti-PD-1/PD-L1 therapy-associated autoimmune blistering disease (13, 14). According to a recently published review, 21 cases of BP have been described in association with PD-1 inhibitors (10 cases were associated with pembrolizumab, 9 cases with nivolumab, one case with durvalumab, and one with atezolizumab) (14). A few other studies have described two cases of atypical PV in patients treated with anti-PD-1 (one related to nivolumab and one with pembrolizumab), one report of PNP with pembrolizumab, and two cases of mucous membrane pemphigoid (MMC) associated with pembrolizumab (15, 16). Among the data presented in our clinical experience, we observed two cases of PB related to PD-1 inhibitor therapy (one case with nivolumab and one with pembrolizumab) (data not published).

Cemiplimab, an immune checkpoint inhibitor (ICI), is a high-affinity potent human immunoglobulin G4 monoclonal antibody capable of inducing programmed cell death. It was approved by the Food and Drug Administration for the treatment of patients with metastatic CSCC or locally advanced CSCC that is not a candidate for surgery or radiation (2).

In this case study we observed that blistering lesions, indicative of bullous disease can occur during cemiplimab therapy for multiple and recurred CSCC. Based on the time of appearance of clinical lesions after initiation of immunotherapy with anti PD-1 and response to steroid therapy, we derived two hypotheses of diagnosis. The first hypothesis is concerned with a PNP revealed by use of cemiplimab therapy, as described in previous literature which states that PNP development has been related to the PD-1 pembrolizumab administration, used for a CSCC (15).

PNP is a severe autoimmune bullous disease, characterised by polymorphous skin lesions involving the mucosa and are associated with benign and malignant neoplasms, such as chronic lymphocytic leukaemia (30.2%), non-Hodgkin lymphoma (26.4%), carcinoma (18.9%), Castleman disease (9.4%), and thymoma (7.5%) (17). The criteria to make a diagnosis of PNP include, the presence of painful mucosal erosions associated with several polymorphous cutaneous eruptions and contemporary serum analysis for the presence of IgG autoantibodies against desmoglein 1 and desmoglein 3 (17). In our case, the absence of polymorphous lesions and mucous involvement, and the positive test for desmoglein 1 rather than desmoglein 3 allowed us to exclude the diagnosis of PNP.

According to the second hypothesis, it is conceivable that a diagnosis of PV without oral mucosal lesions can be induced by PD-1 therapy, whose possibility is confirmed on the basis of desmoglein 1 positivity. In particular, we noted that PV that appeared during cemiplimab administration showed typical manifestation with bullous eruptions, and can also occur at a later time point after the start of immunotherapy (5 months). In the literature, only one case of atypical pemphigus was reported in a patient during immunotherapy with nivolumab administration, for the treatment of urothelial carcinoma (16). Autoimmune skin diseases have been observed in many patients undergoing immunotherapy for cancer. The pathogenetic hypothesis lies in the mechanism of action of these drugs. The onset of autoimmune disease results from the inability to maintain immune self-tolerance. The immune mechanisms that lead to the breakdown of self-tolerance in PV during ICI therapy have not yet been fully elucidated. In order to explain the onset or recurrence of autoimmune diseases during therapy with ICI, it is hypothesised that there is close cooperation between innate immunity, adaptive immunity, T-regulatory, and T-memory cells (18). Furthermore, with particular reference to PV, for the production of anti-desmoglein antibodies, close communication between T and B lymphocytes is necessary. However, the immune mechanisms that lead to the breakdown of tolerance in PV during ICI therapy are not fully understood. From an immunological point of view, it is predicted that both the B and T lymphocyte compartments are involved in PV, or, alternatively, that the autoreactive B cells are widely expressed in patients with PV and ICI therapy can induce the block of tolerance, responsible for the autoimmunity trigger (19). It is also speculated that the antigen presentation of desmoglein by keratinocytes that have many mutations can potentially break tolerance to PV; it is even possible that there might be localised mutations in desmoglein that stimulate loss of tolerance.

The blocking of some pathways by these drugs results in reactivation of the immune system, thus leading to immune activation against skin target proteins by identifying them as antigens. As a confirmation for the above comments, cases of bullous disorders, such as PV and BP, have been reported in the literature after HAART therapy as a manifestation of IRIS, caused by a paradoxical production of auto antibodies against intercellular substances (desmoglein) and dermo-epidermal junction, respectively. The pathogenic mechanism may occur due to aberrant T−cell signalling to B cells, and secretion of immunoglobulin due to the sudden increase in CD4 T−cell count following initiation of HAART (20).

We assumed that cemiplimab immunotherapy could trigger a reactivation of T-cytotoxic immunity, similar to that observed in HIV-infected patients treated with antiretroviral therapy (HAART), in which a real immune reconstitution inflammatory syndrome (IRIS) is observed. We suggest that cemiplimab might have triggered the development of PV in our patient, and blockade of the PD-1/PD-L1 pathway may increase autoantibody production against the desmosomal protein Dsg1, through a process that is both T-cell-and B-cell-mediated.

To our knowledge, this is the first report on an association between PV and cemiplimab therapy. Its peculiarity also lies in the late occurrence of this immune-related adverse event in a very elderly patient. Due to the limited follow-up time and number of patients enrolled in cemiplimab clinical trials, real-world data on immune-related adverse events are becoming increasingly relevant. Of note, despite not being on active therapy, the tumour continued to decrease in size clinically and disappeared completely (**Figure 3**). Of note, for the entire observation period, the patient presented with an absence of bullous manifestation on the skin. In conclusion. During immunotherapy with checkpoint inhibitors, anti-PD-1/anti-PD-L1 autoimmune disorders may occur; however, it is possible to have good control of dermatosis through steroid therapy without negative impact on outcomes.

This case highlights the importance of further studies to improve the understanding of the efficacy as well as skin adverse effect profile of PD-1 inhibitors in elderly patients with advanced CSCC.

## Data Availability Statement

The original contributions presented in the study are included in the article/supplementary material. Further inquiries can be directed to the corresponding author.

## Ethics Statement

Written informed consent was obtained from the patient for the publication of any potentially identifiable images or data included in this article.

## Author Contributions

All authors contributed to the article and approved the submitted version. Conceptualization, RF, and RB. Data collection, RB, VM, RF, DQ, SS and MG. Methodology, RF and MG. Analysis, writing, and editing MG, RB, RF, VM, DQ, and SS. Supervision, RF.

## Conflict of Interest

The authors declare that the research was conducted in the absence of any commercial or financial relationships that could be construed as a potential conflict of interest.

## Publisher’s Note

All claims expressed in this article are solely those of the authors and do not necessarily represent those of their affiliated organizations, or those of the publisher, the editors and the reviewers. Any product that may be evaluated in this article, or claim that may be made by its manufacturer, is not guaranteed or endorsed by the publisher.
